# Evolutionary Insight into the Clock-Associated PRR5 Transcriptional Network of Flowering Plants

**DOI:** 10.1038/s41598-019-39720-2

**Published:** 2019-02-27

**Authors:** Yosuke Toda, Toru Kudo, Toshinori Kinoshita, Norihito Nakamichi

**Affiliations:** 1 Precursory Research for Embryonic Science and Technology, Japan Science and Technology Agency, Kawaguchi, Saitama 332-0022 Japan; 2 Institute of Transformative Bio-molecules, Nagoya University, Furo-cho, Chikusa, Nagoya 464-8602 Japan; 3 Metabologenomics, Inc., 246-2 Mizukami Kakuganji, Tsuruoka, Yamagata 997-0052 Japan; 4 Graduate School of Sciences, Nagoya University, Furo-cho, Chikusa, Nagoya 464-8602 Japan

## Abstract

Circadian clocks regulate the daily timing of metabolic, physiological, and behavioral activities to adapt organisms to day-night cycles. In the model plant *Arabidopsis thaliana*, transcript-translational feedback loops (TTFL) constitute the circadian clock, which is conserved among flowering plants. Arabidopsis TTFL directly regulates key genes in the clock-output pathways, whereas the pathways for clock-output control in other plants is largely unknown. Here, we propose that the transcriptional networks of clock-associated pseudo-response regulators (PRRs) are conserved among flowering plants. Most PRR genes from Arabidopsis, poplar, and rice encode potential transcriptional repressors. The PRR5-target-like gene group includes genes that encode key transcription factors for flowering time regulation, cell elongation, and chloroplast gene expression. The 5′-upstream regions of PRR5-target-like genes from poplar and rice tend to contain G-box-like elements that are potentially recognized by PRRs *in vivo* as has been shown in Arabidopsis. Expression of PRR5-target-like genes from poplar and rice tends to decrease when *PRR*s are expressed, possibly suggesting that the transcriptional network of PRRs is evolutionarily conserved in these plants.

## Introduction

In a wide range of organisms, circadian clocks regulate the daily timing of metabolic, physiological, behavioral, and ecological processes that allow organisms to adapt to environmental changes resulting from day-night cycles. In plants, there are many fundamental processes that are regulated by the clock. In addition, transcriptome analyses for Arabidopsis have demonstrated that genes involved in macro and mineral nutrient uptake, starch synthesis and breakdown, and protection against strong light at sunrise are controlled by the clock^1–3^. Transcriptome analyses for field-grown rice also suggested that the clock contributes significantly to transcriptome regulation in the field^4^. Proper clock function (e.g., proper period length to a given environmental period, proper clock-output gene expression) relates to fitness of Arabidopsis under both laboratory and field conditions^5–8^. In addition, recent molecular-genetic studies revealed that cultivars carrying mutations in genes involved in clock or clock-output events, such as photoperiodic flowering time, were selected as crops were introduced into latitudes or climate areas quite different from their origins^9,10^.

Recent molecular studies revealed that a transcription-translation feedback loop (TTFL) among clock-related transcription factors (TF) underlies the Arabidopsis circadian clock^11,12^. These transcription factors include dawn-expressed CIRCADIAN CLOCK-ASSOCIATED 1 (CCA1) and LATE ELONGATED HYPOCOTYL (LHY), members of the morning-to-evening-expressed PSEUDO-RESPONSE REGULATOR (PRR) family, and evening-to-night-expressed EARLY FLOWERING 3 (ELF3), ELF4, and LUXARRHYTHMO (LUX). ELF3, ELF4, and LUX form a complex named the Evening Complex (EC)^13^. These transcription factors tend to repress genes that are expressed earlier in the day. EC represses *PRR9* and *PRR7*, the PRR family represses *CCA1* and *LHY*, and CCA1 and LHY repress *PRR*s, *ELF4* and *LUX*. In addition to these repressors, the REVEILLE family (RVE) and the NIGHT LIGHT-INDUCIBLE AND CLOCK-REGULATED (LNK) family directly induce *PRR5* and *TOC1*, two members of the *PRR* family^14,15^. These transcription factors are highly conserved among angiosperms at the protein sequence level^16^. In addition, a rice *PRR7* homologue (*OsPRR37*) and a *TOC1* homologue (*OsPRR1*) complimented the Arabidopsis *prr7* and *toc1* mutants, respectively, strongly suggesting that these *PRR* family functions are conserved in angiosperms^17^.

The PRR family, CCA1, and EC directly regulate the expression of genes implicated in clock-output pathways. CCA1 directly regulates drought-stress responses, abscisic acid signaling, and brassinosteriod signaling^18–20^. The PRR family directly regulates genes encoding transcription factors involved in cold-stress responses, cell elongation, and repression of flowering^21–24^. EC directly controls photosynthesis, temperature responses, ethylene signaling, cell elongation and cytokinin signaling^13,25^. These results indicate that the clock regulates a wide range of physiological responses by regulating the expression of genes, especially those encoding transcription factors. Despite the sequence similarities of clock-associated genes among angiosperms, the molecular connections between the clock TTFL and output pathways in plants other than Arabidopsis are largely unknown.

Arabidopsis *PRR* genes were named due to their similarity with bacterial response regulator proteins that play roles in output functions in signal transduction pathways^26^. Although authentic response regulators in plants receive a phosphoryl group from sensory histidine kinase and Hpt proteins in cytokinin signal transduction, PRRs are not likely involved in cytokinin signaling. PRRs cannot receive a phosphoryl group from sensory kinases^26^ because the key Asp residue that accepts a phosphoryl group from sensory kinases is not conserved in the pseudo-receiver (PR) domain in the N-termini of PRRs. The PR domain of PRRs is involved in protein-protein interaction. Both PRR3 and PRR5 bind TIMING OF CAB EXPRESSION 1 (TOC1, the same gene as PRR1) through the PR domains^27,28^. PR domains of PRR5 and TOC1 are bound by ZEITLUPE (ZTL), an F-box domain-containing protein that trigger degradation of PRR5 and TOC1^29,30^.

A CONSTANS, CONSTANS-LIKE, and TOC1 (CCT) domain is at the C-termini of PRRs and is involved in protein-protein interaction and protein-DNA interaction. The domain is essential for recognition of target genes by PRRs^21,31^. CCT from non-functional alleles of TOC1 (*toc1-1*) and that from PRR3 have reduced abilities to associate with the promoter region of *CCA1*, a typical PRR-target gene, suggesting that CCT is involved in target DNA recognition^21,31^.

Repression motifs of PRR9, PRR7, and PRR5 lie between the PR and CCT domains and consist of two conserved regions, L(^E^/_D_)(^L^/_I_)S(^L^/_I_)(^R^/_K_)R and SXXSAF(^S^/_T_)(^R^/_Q_)(^Y^/_F_)^32^. The repression motif for these three PRRs binds to TOPLESS/TOPLESS-RELATED (TPL/TPR), members of the plant Groucho/Tup1 co-repressor family^33^. Thus TPL/TPR associates with *CCA1* and *LHY*, two PRR-target genes, and causes histone deacetylation and repression of these genes. From the deduced function of these domains, Arabidopsis PRR (PRR9, PRR7, and PRR5) are proposed to act as transcriptional repressors. Since repression activity of TOC1 *in vivo* is in the PR domain, it is possible that TOC1 could regulate transcription when TOC1 binds to transcription factors^31^.

In this study, we report that all PRR proteins from Arabidopsis, poplar, and rice act as possible transcriptional repressors, except for Arabidopsis PRR3 and rice PRR59. In addition, expression profiles suggest that homologues of the Arabidopsis PRR5-target genes in rice and poplar are also likely regulated by PRRs. Homologues of *PIF4* (*Phytochrome Interacting Factor 4*)/*PIF5*, key transcription factors involved in red-light signaling^34^, *B-BOX DOMAIN PROTEIN* (*BBX*) family^35^ homologues involved in light signaling and flowering time regulation, *CYCLING DOF FACTOR* (*CDF*) family^36^ homologues involved in flowering time, and *SIGMA FACTOR E* (*SIGE*)^37^ involved in gene expression in chloroplasts, are expressed at a time when *PRR* genes are rarely expressed. Collectively, these results may suggest that the PRR-transcriptional network controlling these key transcription factors is conserved in flowering plants.

## Results

### Comparisons among three PRR protein domains

**Figure 1:**
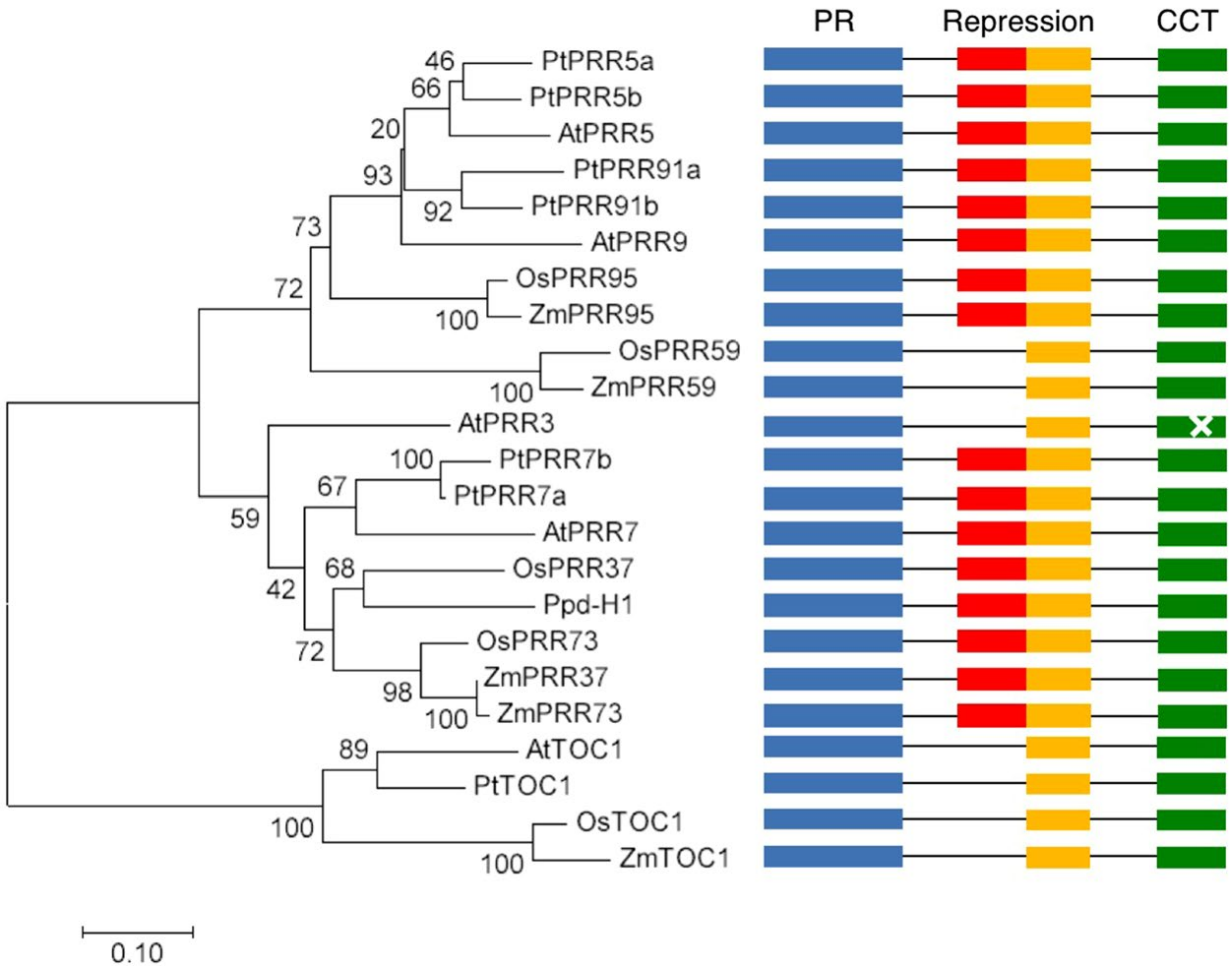
Phylogenic tree of Arabidopsis, poplar, and rice PRR proteins. A phylogenic tree was inferred by the neighbor-joining method. Protein sequences of pseudo-receiver (PR) domains of Arabidopsis PRR (AtPRR or AtTOC1), poplar PRR (PtPRR or PtTOC1), rice PRR (OsPRR), maize PRR (ZmPRR or ZmTOC1), and a barley PRR (Ppd-H1) were used to construct the phylogenic tree. Domains in PRR proteins are represented in bars of different colors: PR (blue), repression domain (Repression, red and yellow), and CONSTANS, CONSTANS-LIKE1, TOC1 (CCT) domain (green). A point mutation in the CCT domain in AtPRR3 resulted in weak recognition of target genes compared to other Arabidopsis PRR proteins.

To understand whether PRRs from other plants also function as transcriptional repressors, we compared the domains of PRR orthologues from Arabidopsis, poplar, rice, maize, and a barley PRR (Ppd-H1) (Fig. 1). Phylogenic analysis using the PR domain, the longest domain found in all PRRs examined in this study, demonstrated that the PRR family can be classified into three sub-groups (the TOC1 group, the PRR9/PRR5 group, and the PRR7/PRR3 group) as reported by a previous study (Fig. 1)^38^. Although the PR and CCT domains are found in all PRRs examined in this study, full-length repression domains were found only in the PRR9/PRR5 and PRR7/PRR3 sub-groups, except for AtPRR3, OsPRR59, and ZmPRR59 (Fig. 1 and Supplementary Fig. 1). These results suggested that proteins belonging to the PRR9/PRR5 and PRR7/PRR3 groups, except for AtPRR3, OsPRR59 and ZmPRR59, act as transcriptional repressors. Given that the PR domain of AtTOC1 has repression activity *in vivo*, likely due to binding to other PRRs or different types of TF^31,39^, we hypothesize that TOC1 orthologues in poplar and rice also act as transcriptional repressors despite lacking the repression domain.

### Expression of PRR genes

**Figure 2:**
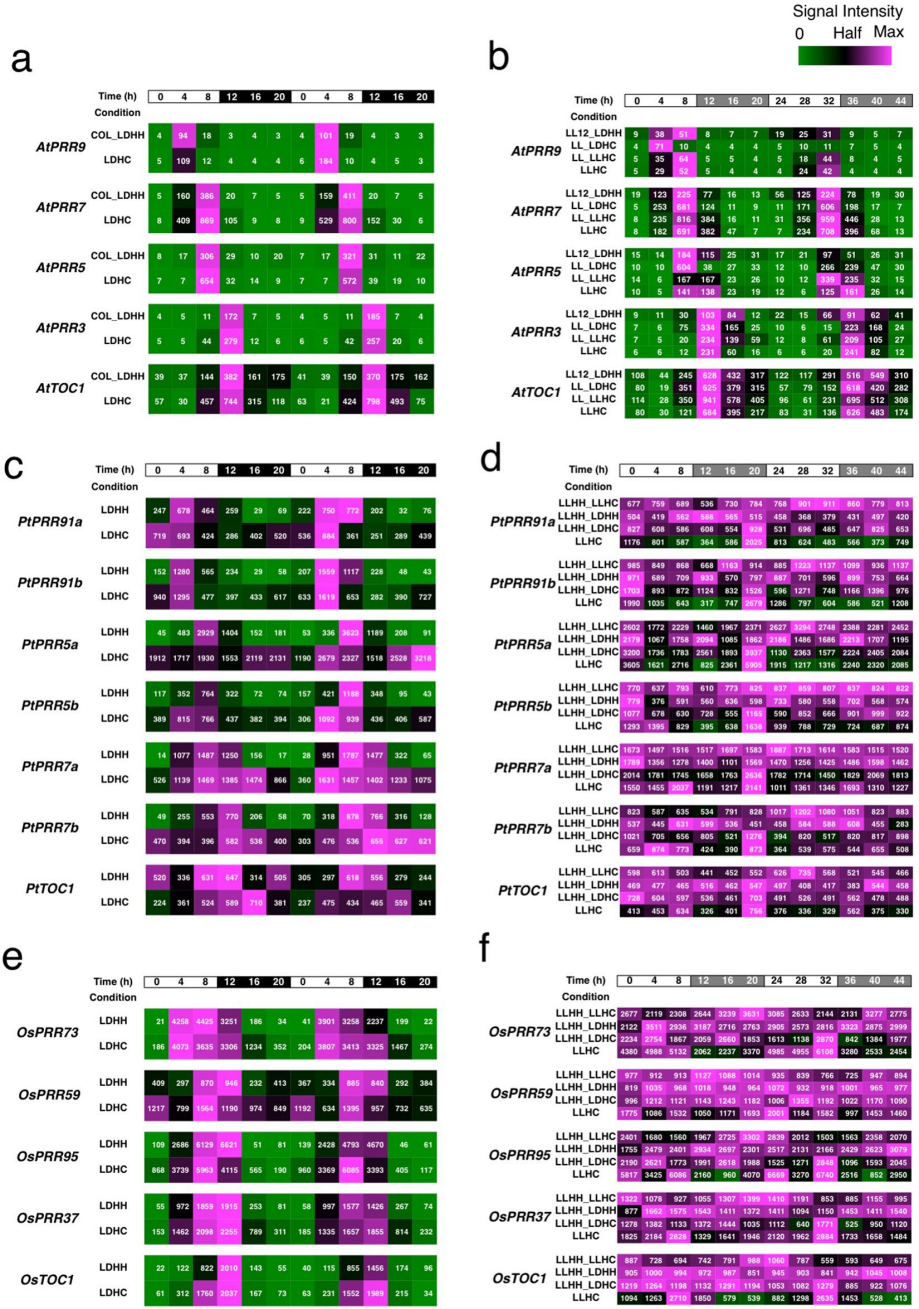
Expression of *PRR* genes in Arabidopsis, poplar, and rice. Expression of *PRR* genes in Arabidiosis (**a**,**b**), poplar (**c**,**d**), and rice (**e**,**f**) was surveyed in the public microarray database Diurnal. Signal intensities are visualized in a heat-map. The maximum expression of each gene at specific light conditions is identified as a magenta block. One-half of the maximum expression is denoted as a black block, and zero expression by a green block. The left panel summarizes experiments conducted under light-dark conditions, and the right panel shows heat maps from constant light conditions. Growth conditions were shown in detailed in Supplementary Table 1.

To characterize and compare the expression patterns of *PRR* genes in Arabidopsis, poplar, and rice, we surveyed the publicly available database, Diurnal 2.0 (http://diurnal.mocklerlab.org)^40^. As reported in many studies, *AtPRR9*, *AtPRR7*, *AtPRR5*, *AtPRR3*, and *AtTOC1* are expressed sequentially from early morning to evening under diurnal light-dark conditions (Fig. 2a and Supplementary Fig. 2). The sequential expression patterns of Arabidopsis *PRR* genes were also detected under constant light conditions including constant dark, showing that expression of *PRR* genes is under clock control (Fig. 2b and Supplementary Fig. 2).

We next surveyed the expression of poplar *PRR* genes in the Diurnal database (Fig. 2c,d). Expression of poplar *PRR* is cyclic under light-dark cycle conditions. Peak expression of poplar *PRR* occurred from early morning to evening, except for *PtPRR7b* expression during the night under LDHC conditions (12 h light hot - 12 h dark cold). Expression peaks for *PtPRR91a* and *PtPRR91b* occurred in the morning, those for *PtPRR5a*, *PtPRR5b*, *PtPRR7a* and *PtPRR7b* occurred in the afternoon, and expression of *PtTOC1* was highest in the evening under light-dark conditions. However, the expression of *PtPRR* genes was nearly constant under constant light conditions (Fig. 2d). To judge whether constant expression under constant light conditions was a *PtPRR*-gene-specific phenomenon, expression of other clock-associated genes (e.g., *PtLHY1*, *PtLHY2*, *PtGI*, and *PtGIL*) was surveyed^41,42^. Expression of *PtLHY1*, *PtLHY2*, *PtGI*, and *PtGIL* was rhythmic under light-dark conditions but relatively constant under constant light conditions (Supplementary Fig. 3). These results suggested that the expression of poplar diurnally-cyclic genes is no longer cyclic under constant light conditions used in the Diurnal database.

Next, we surveyed the expression of rice *PRR* genes (*OsPRRs*) in the Diurnal database. The expression of rice *TOC1/PRR* genes showed a diurnal rhythm under light-dark conditions as previously demonstrated (Fig. 2e). All *OsPRR* genes were expressed from noon to early night under light-dark conditions. However, under constant light conditions, the expression of *OsPRR* genes was relatively constant as previously reported^38^ (Fig. 2f). We also found low-amplitude expression of homologues of *LHY* and *GI* (*OsLHY* and *OsGI*, respectively)^43,44^ under constant conditions (Supplementary Fig. 3), suggesting that the amplitude of circadian rhythms of rice under constant light conditions was lower than in Arabidopsis.

Collectively, all *PRR* genes in Arabidopsis, poplar, and rice were expressed in a diurnal rhythmic pattern during the day. The timing of expression for each *PRR* was unique in each plant, suggesting that a sequential expression pattern of poplar and rice *PRRs* may be crucial for clock function as reported in a study using Arabidopsis^32^.

### Identification of AtPRR5-target-like genes in poplar and rice

To examine whether the transcriptional regulation of PRR is conserved in other plants, homologous genes of the Arabidopsis PRR5-targets were identified in poplar and rice and the expression of these genes was analyzed. The strategy for analyzing a potential PRR-transcription network in this study is shown in Supplementary Fig. 4. The two top-ranked genes having sequence similarity to the AtPRR5-target genes^21^ were identified from the poplar and rice genomes (https://phytozome.jgi.doe.gov/pz/portal.html) (Supplementary Tables 2 and 3). Most of the AtPRR5-target genes had at least two similar genes in poplar. *AT1G14280* (*PKS2*, *PHYTOCHROME KINASE SUBSTRATE 2*) and *AT2G23120* (unknown function) had sequence similarity to only one poplar gene. Some poplar genes were multiply hit by distinct AtPRR5-targets. Similarities between an AtPRR5-target gene and a possible homologous gene in poplar ranged from 26.7 to 97.1% with a mean and median of 66.1 and 66.9%, respectively (Supplementary Table 2).

Rice genes homologous to the AtPRR5-targets were identified from the Phytozome database. We obtained the top two rice genes with sequence similarity to AtPRR5-targets but did not find rice genes having similarity to *Arabidopsis PKS2*, *AT1G68440*, *AT1G69160*, *AT2G23120*, or *LNK4*. Single rice genes with similarity to *LHY*, *CCA1*, and *LNK2* were found. As in the case with poplar, some rice genes were multiply hit by different AtPRR5-targets (Supplementary Table 3). Similarities between AtPRR5-target genes and possible homologous genes in rice ranged from 20.2 to 96.1% with a mean and median of 55.1 and 55.5%, respectively (Supplementary Table 3).

### Enriched DNA sequences in the 5′-upstream region of AtPRR5-target-like genes

**Figure 3:**
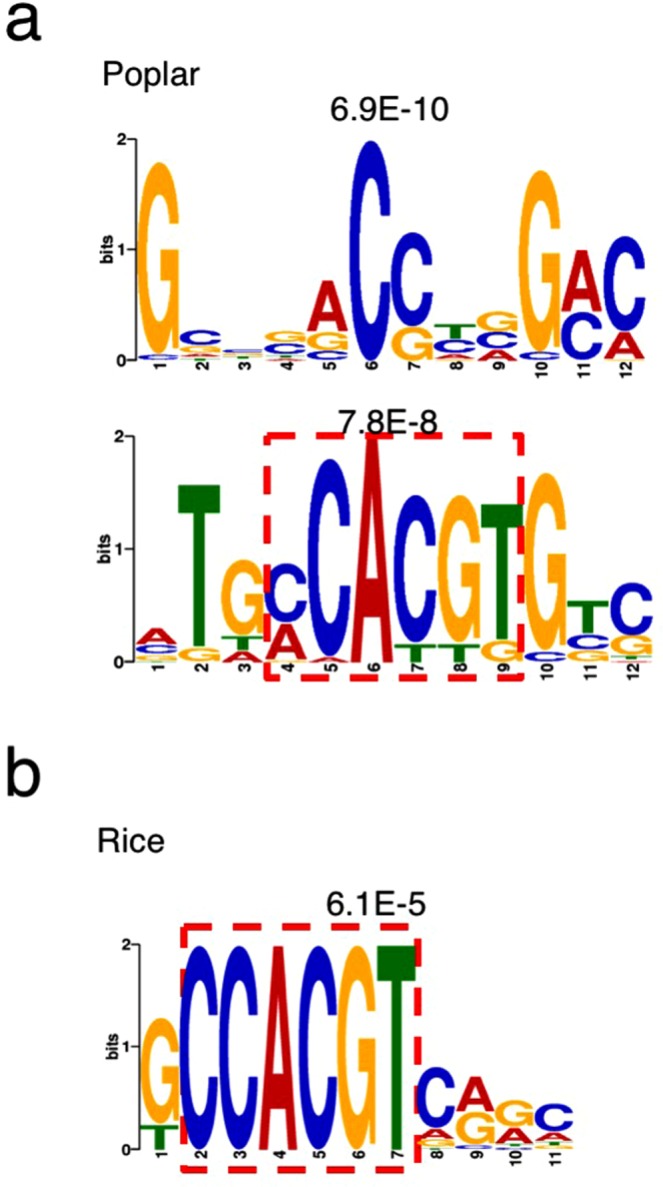
MEME (Multiple Em for Motif Elicitation) analysis of 5′-upstream regions of AtPRR5-target-like genes. Common motifs found in the 5′-upstream regions of PRR5-target-like genes in poplar (**a**) and rice (**b**). Non-repeated sequences are shown. Repeated sequences are shown in Supplementary Fig. 5. The areas surrounded by red dashed lines denote G-box-like sequences.

Although AtPRR has not been demonstrated to directly bind to G-box-like sequences *in vitro*, the 5′-upstream regions (possible promoter regions) of AtPRR5-, AtPRR7-, and AtPRR9-target genes contain G-box-like (CACGTG) sequences^21,23,24^. In addition, the G-box-like sequence of *CCA1* is necessary for transcriptional control by AtPRR7 and AtPRR9^24^, suggesting that a G-box-like sequence is crucial for PRR-dependent transcriptional regulation possibly with other proteins that directly recognize G-box-like sequences. To examine whether poplar PRRs recognize the upstream region of AtPRR5-target-like genes, 2.0 kb 5′-upstream regions of AtPRR5-target-like gene coding sequences were analyzed by MEME (Multiple Em for Motif Elicitation, http://meme-suite.org) (Supplementary Dataset 1, Fig. 3, and Supplementary Fig. 5). The analysis revealed that GCBSACCYVGMC (6.9 e-10), MTGMCACGTGTC (7.8 e-8) and some repeated sequences (e.g., AAAA, ATATAT) are enriched in the 5′-upstream region of poplar AtPRR5-target-like genes (Fig. 3a, Supplementary Fig. 5). The MTGMCACGTGTC sequence contains a typical G-box, suggesting that poplar PRR may interact with the sequence to control expression as in *Arabidopsis*.

MEME analysis for rice revealed that some repeated sequences (e.g., GGGG, AAAA, ATATAT), and GCCACGTCRRC (6.1 e-5) are enriched in the 5′-upstream region of the AtPRR5-target-like genes in rice (Fig. 3b, Supplementary Fig. 5, Supplementary Dataset 2). The GCCACGTCRRC sequence contains a G-box-like motif, suggesting that rice PRR homologues may recognize G-box-like sequences upstream of rice AtPRR5-target-like genes *in vivo*.

### Expression pattern of AtPRR5-target-like genes

**Figure 4:**
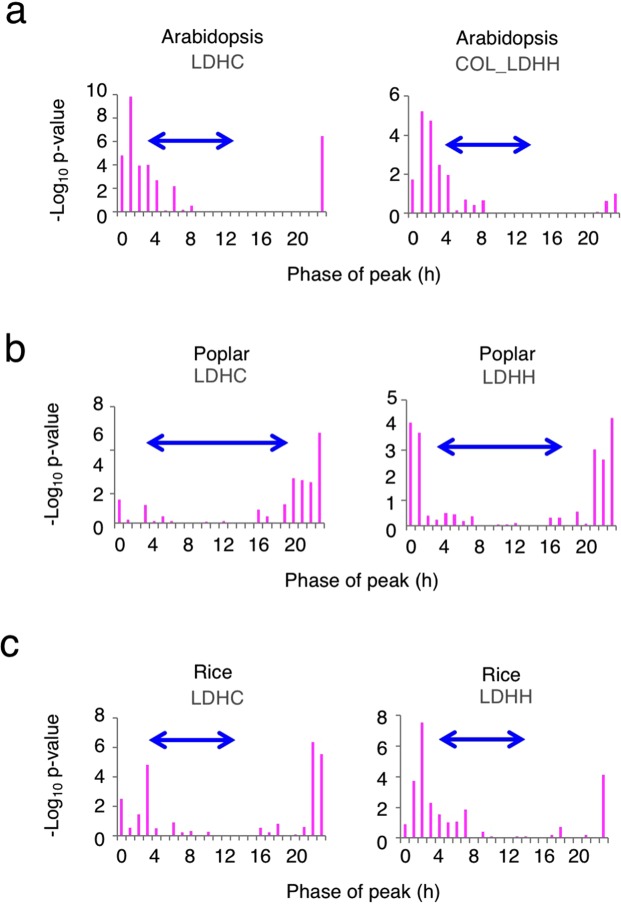
Phase enrichment of expressed AtPRR5-target-like genes in Arabidopsis, poplar, and rice. (**a**) PRR5-target genes in Arabidopsis were analyzed by Phaser (LDHC condition and COL_LDHH condition). Phase enrichment of PRR5-target-like genes in poplar (**b**) (LDHC and LDHH), and in rice (**c**) (LDHC and LDCC). Blue arrows indicate the time when *PRR* genes are expressed as described in the legend to Fig. 2.

Previous studies demonstrated that AtPRR9, AtPRR7, AtPRR5, and AtTOC1 share target genes and repress the expression of targets during the day^21–24^. Expression of AtPRR5-targets was cyclic with highest expression from dawn to early morning in Arabidopsis (Fig. 4a, under two light-dark conditions, LDHC and COL_LDHH, as analyzed in Phaser with a correction cutoff value of 0.7), a result that is consistent with our previous study^21^.

To determine the expression patterns of poplar AtPRR5-target homologues, gene expression was analyzed in two light-dark conditions (LDHC and LDHH as analyzed in Phaser with a correction cutoff value of 0.7, Fig. 4b). The Phaser analyses indicated that there was enriched, rhythmic expression of poplar AtPRR5-target-like genes. The expression peaks of these poplar genes were detected around dawn and early morning, a pattern resembling the expression pattern of Arabidopsis PRR5-targets^21^. Phaser analyses indicate that there is enrichment of rhythmically expressed AtPRR5-target like genes when rice is grown under two light-dark conditions (Fig. 4c). The Phaser analyses also indicated that AtPRR5-target homologues in rice are expressed around dawn, a similar expression pattern as that seen for Arabidopsis PRR5-targets.

### Detailed expression pattern of PRR5-target-like genes encoding transcription factors

**Figure 5:**
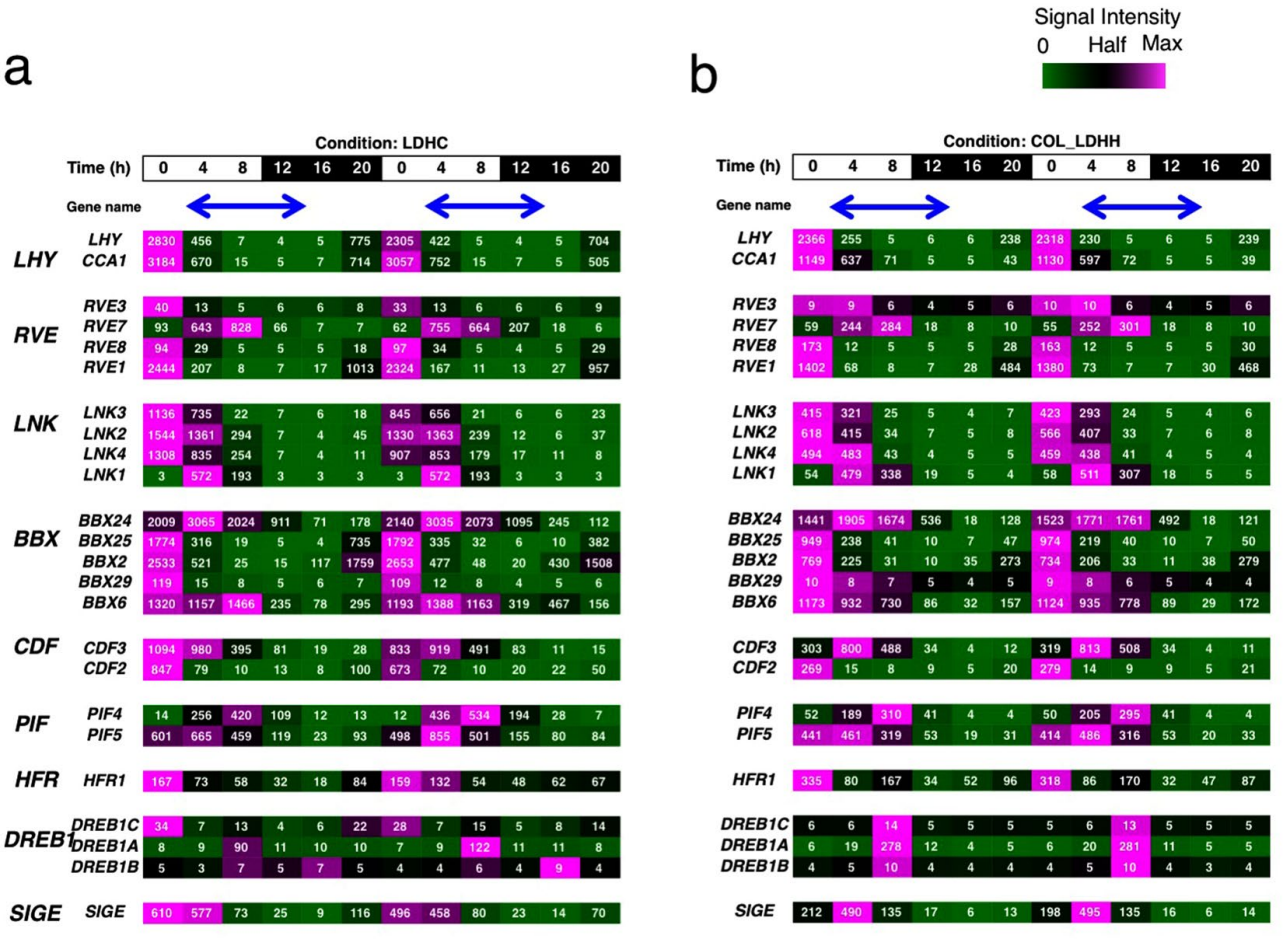
Expression of AtPRR5-target genes encoding transcription factors. Expression of PRR5-target genes encoding transcription factors in Arabidopsis was surveyed using Diurnal (LDHC and COL_LDHH conditions are shown in panels a and b, respectively). Expression values are visualized as heat maps as described in the legend to Fig. 2. Blue arrows indicate the time and duration when *PRR* genes were expressed.

Among the AtPRR5-target genes, genes encoding transcription factors play a critical role in the timing regulation of clock-output pathways^21^. To understand whether PRR transcription networks govern similar output regulation in poplar and rice, we further focused on AtPRR5-target-like genes that encoded transcription factors. These transcription factors included six members of the MYB family [CCA1, LHY, EARLY PHYTOCHROME RESPONSIVE 1 (EPR1)/also called REVEILLE7 (RVE7), RVE1, RVE3, and RVE8], three members of the DOF family [CDF2, CDF3, and CDF5], five members of the C2C2-CO–like family [BBX2, also known as CONSTANS-LIKE 1 (COL1)], BBX6/COL5, BBX24/SALT TOLERANCE (STO), BBX25/STH (SALT TOLERANCE HOMOLOGUE), and BBX29]], three members of the bHLH family [PIF4, PIF5, and LONGHYPOCOTYINFAR-RED (HFR1)], three members of the AP2/EREBP family (DEHYDRATION-RESPONSIVE ELEMENT BINDING 1 [DREB1, also called C-REPEAT BINDINGFACTOR (CBF) DREB1A, DREB1B, and DREB1C], SIGMA FACTOR E (SIGE, also called SIG5), and three members of the PRR family (PRR9, PRR7, and PRR5). The biological functions of AtPRR5-targets were described previously^21^. Briefly, CCA1, LHY, RVE, and PRRs are involved in clock function. BBX2/COL1 may have a clock-related function, CDFs and BBX6/COL5 function in flowering time regulation, and BBX24/STO is involved in light signaling. PIF4 and PIF5 are involved in hypocotyl elongation in the dark, whereas HFR1 represses hypocotyl growth in the light. Three DREB1 proteins regulate cold-stress responses. SIGE controls gene expression in chloroplasts. Expression of these transcription factor-encoding genes was cyclic with peaks from dawn to noon (Fig. 5), except for *DREB1B* (*At4g25490*) whose expression peaked during the night under light hot/dark cold conditions, as reported in previous studies^12,41^.

**Figure 6:**
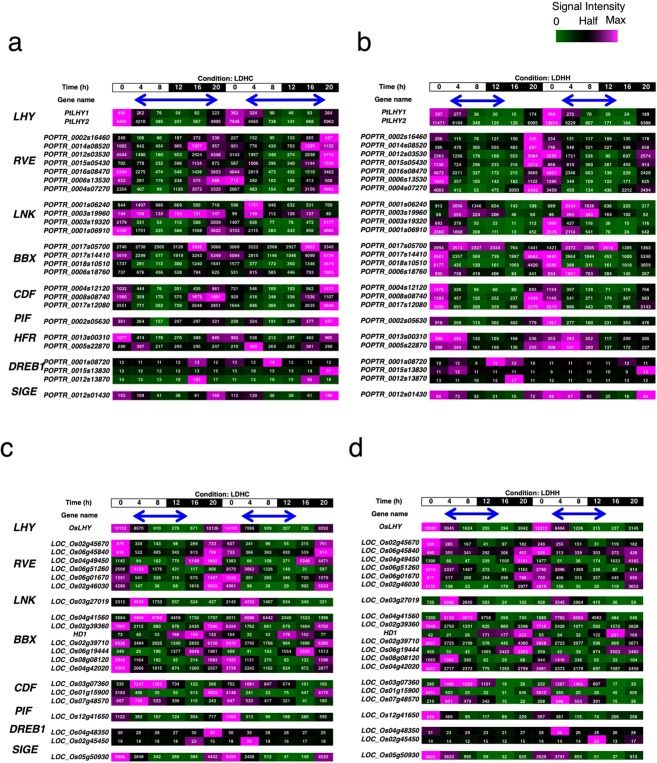
Expression of AtPRR5-target-like genes encoding transcription factors in poplar and rice. Expression of PRR5-target-like genes encoding transcription factors in poplar was surveyed using Diurnal (LDHC and LDHH in poplar are shown in panels a and b. LDHC and LDHH in rice are shown in panels c and d). Blue arrows indicate the time and duration when *PRR* genes were expressed.

To understand whether the PRR5-transcriptional network in Arabidopsis is conserved in poplar, the expression of poplar orthologues of these transcription factor-encoding genes was analyzed. Peak expression of poplar *LHY*, *RVE*, *CDF*, *PIF*, *HFR* and *SIGE* homologues occurred from dawn to noon (Fig. 6a,b). The *CDF* expression pattern was consistent with a previous study^42^. Except for one *LNK* homologue under LDHC conditions, the *LNK* genes were mostly expressed from dawn to noon. One *BBX* homologue was expressed from the morning to evening under LDHH conditions and during the late night under LDHC conditions. The other *BBX* homologues were expressed at around dawn. The expression of *DREB1* homologues was mostly constant under LDHH conditions and overall expression increased during the cold nights of LDHC conditions. Collectively, most poplar AtPRR5-target-like genes encoding TF were repressed when *PtPRR* genes were expressed. Given that poplar PRR homologues feature domains essential for transcriptional repressors (Fig. 1), it is possible that poplar PRR negatively regulates the expression of these genes during the day.

In rice, *LHY*, *RVE*, *LNK*, *CDF*, *PIF*, and *SIGE* homologues were expressed from late night to noon (Fig. 6c,d). *BBX* homologues were expressed cyclically with peaks at various phases. Two *BBX* genes were expressed during the night, four were expressed at around dawn, and one was expressed during the morning under LDHC conditions. Similar expression peaks of these *BBX* homologues were found under LDHH conditions. Expression of *DREB1* homologues was too low to measure under both light-dark conditions. The majority of the AtPRR5-target gene homologues encoding TF in rice were expressed before the rice *PRR* genes were expressed, suggesting that rice PRR downregulates the expression of these genes.

**Figure 7:**
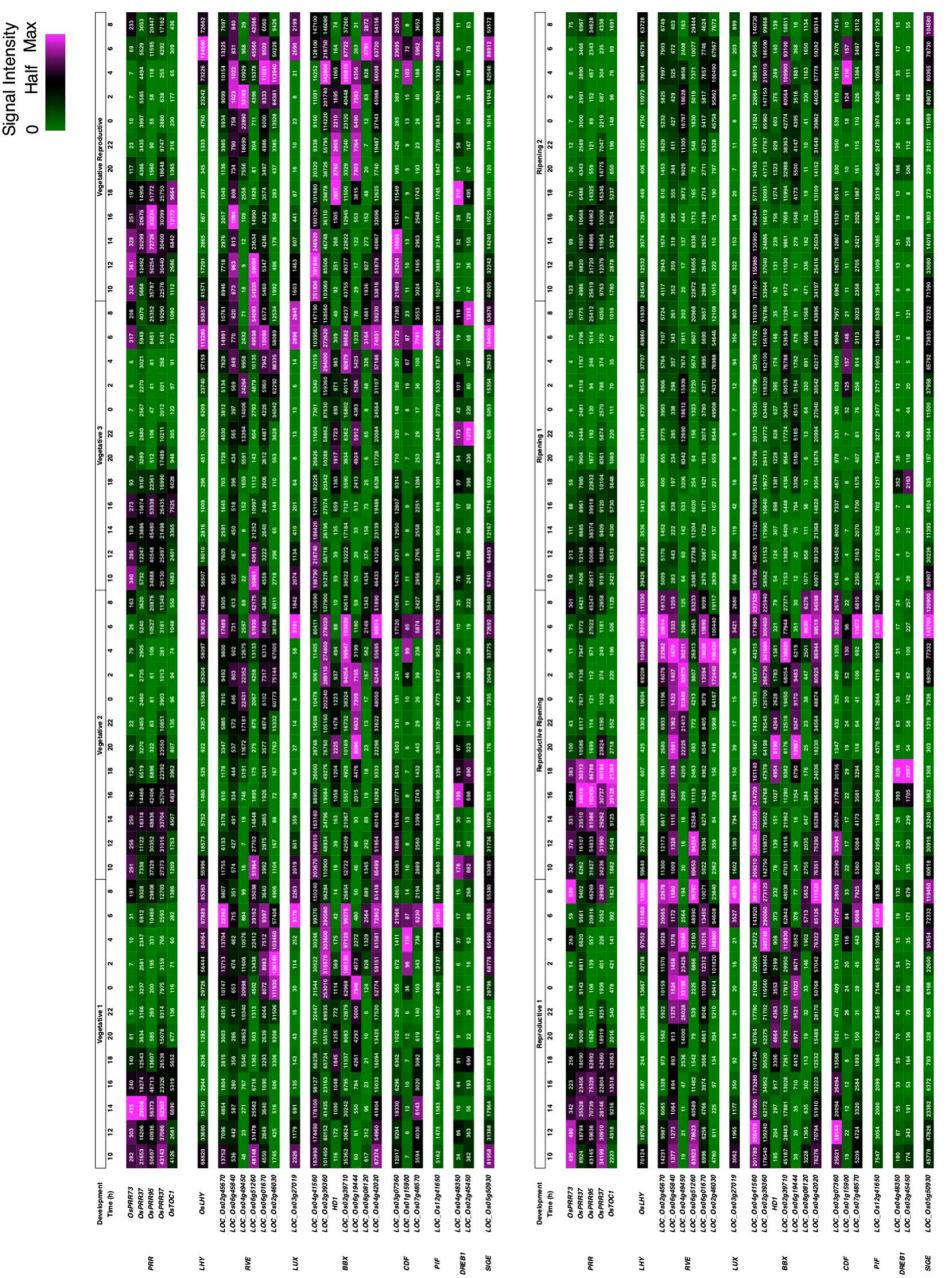
Expression of AtPRR5-target-like genes encoding transcription factors in rice grown in field conditions. Expression of rice AtPRR5-target-like genes encoding transcription factors was surveyed using RiceXPro (http://ricexpro.dna.affrc.go.jp). Expression values are visualized as heat maps as described in the legend to Fig. 2. Developmental stages are shown in white boxes as described in RiceXPro.

We next examined the expression of AtPRR5-target-like TF under field conditions^4^ to evaluate how environmental signals, including unknown cues, potentially affect the PRR transcriptional network. *OsPRR* expression was found during the day (exact time 08:00 h to 18:00 h) during vegetative, reproductive, and ripening phases of development (Fig. 7). The expression of most AtPRR5-target TF showed a diurnal pattern, except for the *DREB1* homologues. Among these rhythmically-expressed genes, only a *BBX* homologue (*LOC_Os04g41560*) and a *CDF* homologue (*LOC_Os03g07360*) had expression peaks during the day when *OsPRRs* were highly expressed. The expression of the other 18 genes was found to occur when *OsPRRs* were barely expressed, suggesting that *OsPRRs* negatively control the expression of these genes even under field conditions.

## Discussion

The plant circadian clock is a time-keeping system that governs the rhythmic expression of many physiological processes. Molecular studies have demonstrated that photoperiodic flowering time regulation and diurnal hypocotyl growth are crucial clock outputs^34^. In addition, expression of cold-stress responses at certain times of the day controlled by the clock mechanism seem important for adaptation to the environment^45^. Furthermore, metabolic regulation is also regulated by the clock^3^. Transcriptome analyses of Arabidopsis, poplar, and rice demonstrated that genome-wide rhythmic gene expression is commonly observed in these plants^2,46^. A combined systems-biology approach (chromatin immunoprecipitation-coupled with deep sequencing, transcriptome analyses using clock mutants, transgenic lines with over- or inducibly expressed clock-associated transcription factors, including rationally designed, synthetic clock transcription factors) in Arabidopsis revealed the details of the clock’s transcriptional network^8,18,19,21–25,31^. However, our understanding of the evolutionary perspectives of the transcriptional network underpinning the clock is still poor.

As a starting point toward understanding the entire clock transcriptional network in an evolutionary scenario, we examined whether the AtPRR5 transcriptional network is conserved in a model dicot and a model monocot. Our initial observations revealed that both poplar and rice proteins from the PRR9/PRR5 and PRR7/PRR3 sub-groups, except for OsPRR59, have three common domains, namely the PR, repression, and CCT domains (Fig. 1). The repression and CCT domains are involved in transcriptional repression and target DNA recognition, respectively, suggesting that these PRRs are transcriptional repressors. Our analysis found that G-box-like sequences are enriched in poplar and rice AtPRR5-target-like genes. Thus, PRRs may bind to G-box-like sequences in these plants; however, we hypothesize that PRRs are more likely to recognize G-box-like sequences through various protein-protein interactions^32^. Indeed, the PRR family, including TOC1, can bind to bHLH transcription factors (PIF family)^47,48^ that directly recognize G-boxes, and PRR regulates PIF-target genes^48–50^. The exact molecular mechanism for DNA-recognition of major PRR5-target genes, however, remains unknown. We tested At-PRR5 target genes in the Ara-BOX-cis database (araboxcis.org)^51^ to examine whether known bZIP and bHLH transcription factors are involved in PRR5 transcriptional regulation and found that a minor population of AtPRR5-target genes are bound by known bZIP and bHLH transcription factors (Supplementary Fig. 6). Thus, it is possible that PRR5 and these bZIP or bHLH transcription factors may function in transcription regulatory complexes to regulate some AtPRR5-targets, but another unknown mechanism plays a more major role in regulating the PRR5-transcription network.

Rice and poplar TOC1 orthologues also have PR and CCT domains but are missing the repression domain as is present in Arabidopsis TOC1. Despite the loss of the repression domain, Arabidopsis TOC1 has repression activity through the PR domain that may interact with other transcriptional regulators such as PRR5 or other transcription factors *in vivo*^31^. TOC1 orthologues in other plants may function as proteins in a transcriptional regulator complex. OsPRR59 may act in the clock system by binding to other PRR family members or transcription factors through its PR domain as in the case of AtPRR3^27^. Interestingly, overexpression of *AtPRR3* resulted in opposite clock-output phenotypes (late flowering and long hypocotyls) compared to those of *AtPRR9*, *PRR7*, and *PRR5* (early flowering and short hypocotyls)^52^.

Our study demonstrated that possible orthologues of Arabidopsis PRR5-targets in poplar and rice are expressed from late night to morning and are suppressed from noon to midnight, coincident with the time when poplar and rice *PRR* genes are expressed (Figs 4 and 6) as reported previously in Arabidopsis^21^. Therefore, PRR proteins possibly downregulate the expression of some PRR5-target orthologues in poplar and rice from noon to midnight.

**Figure 8:**
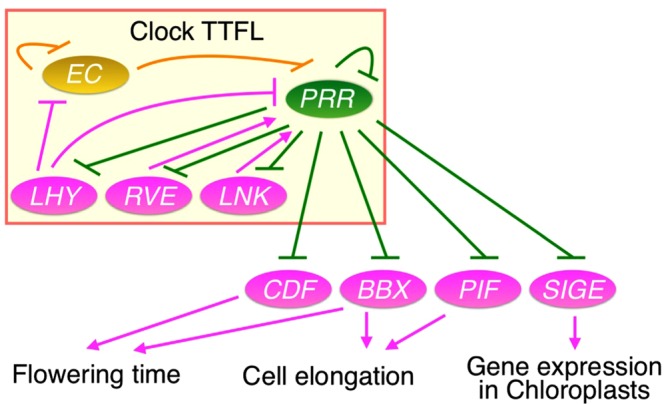
A possible model of the PRR transcription network in flowering plants. PRRs regulate the *LHY*, *RVE*, and *LNK* genes in the clock TTFL. PRRs also regulate *BBX*, *CDF*, *PIF*, and *SIGE* as clock-output controls. Multiple genes exist within each gene family. Possible targets of PRR are expressed from dawn to morning.

Possible AtPRR5-targets in poplar and rice include *LHY*, *RVE*, *LNK*, *CDF*, *PIF*, *BBX*, and *SIGE*, all genes that encode transcription factors (Fig. 8). *LHY*, *RVE*, and *LNK* are involved in the clock TTFL, and expression of these genes occurs from dawn to morning in Arabidopsis^12^. Homologues of these TF genes in rice and poplar were also expressed from dawn to noon, at a time when *PRR* genes are less expressed (Fig. 6). These results suggested that the negative regulation of these genes by PRR proteins is conserved in the clocks of Arabidopsis, poplar, and rice. CDF is a key transcription factor involved in flowering time regulation by repressing the transcription of *CONSTANS* (*CO*) and *FT* in the photoperiodic flowering pathway in Arabidopsis^36,53^. Our results suggest that poplar and rice PRRs may also repress *CDF* to regulate flowering time (Fig. 5). Such repressive action of PRR on *CDF* expression was observed in field-grown rice (Fig. 7), suggesting a relationship that is quite robust against environmental fluctuations. It should be noted that the effects of CDF on flowering induction are opposite in long-day plants (Arabidopsis and pea) and a short-day plant (rice)^36,54,55^. This observation is likely due to the opposite effect of *CO*, a target gene of CDF, on the transcription of *FT*^56^. Nevertheless, our analysis proposes the possible transcriptional regulation of *CDF* by PRR in the three plants. In addition, since *PRR* genes are involved in flowering time regulation in divergent plants (Arabidopsis, rice, barley, wheat, pea, sorghum, and sugar beet)^9^, it is possible that PRR also regulates *CDF* in these plants, since *CDF* expression is suppressed when *PRR* is expressed in three divergent angiosperm plants (Figs 5, 6 and 7). Although *PRR* and *CDF* have not been reported to be involved in flowering regulation in poplar, the CO/FT module controls photoperiodic flowering^57^. Given that the circadian clock is a timekeeping system for photoperiodic flowering time in many plants, including trees, it is not surprising for PRR, the clock-transcriptional repressor, to regulate the CO/FT module and flowering time via control of *CDF* transcription in poplar.

In addition to the transcriptional repressive function of PRRs (PRR9, PRR7, PRR5, and TOC1), other molecular functions such as stabilization of CO by protein-protein interactions has been emerging as a critical factor for photoperiodic flowering time^58^. Dual *PRR*s functions on clock control and flowering time regulation in the dicot Arabidopsis may provide new insight into the molecular function of PRR73/37 in monocots, since some members of the monocot *PRR73/37* family were regarded to regulate flowering time but not clock control^59–61^. Our study used transcriptome data from whole plants^46^. The interaction between PRR and CO should occur in vein tissues where CO is expressed^58^. The proportion of transcripts from vein tissues is relatively low among transcripts isolated from whole plants^62^, suggesting that our analysis was unlikely to detect more than a trace of transcript changes due to PRR-CO interaction.

We found a possible negative regulation of *PIF* by PRR in poplar and rice (Figs 6 and 7). Though *PIF* transcription is sensitive to light and temperature^63^, and *PIF* is also repressed by EC^13^, *PIF* expression is always low when *PRRs* are expressed in the field where light and temperature fluctuations occur (Fig. 7). This observation may suggest that negative regulation of *PIF* by PRR is robust against environmental fluctuations. The interaction of PRR and EC for *PIF* transcription is a subject for future consideration. Physical interaction between PRR and PIF proteins can modulate expression of PIF-targets^48–50^. We did not observe upregulation of *HFR*, one of the PIF- and PRR-target genes, when *PRRs* were expressed in *Arabidopsis* and poplar (Fig. 6). Since the PIF protein is also sensitive to light signals^33^, external signals may mask PRR protein function on PIF activity.

*SIGE* expression is controlled by the clock, is transcribed from nuclear genomic DNA, and encodes a sigma factor that regulates transcription of genes encoded in chloroplast DNA^37^. This gene encodes the only protein in the *SIG* family that is controlled by AtPRR5 and AtPRR7 and is expressed around dawn^21^. Diurnal expression of *SIGE* is crucial for not only diurnal expression of some chloroplast genes but also for the time-dependent gene induction by blue light^37^. *SIGE* homologues in poplar and rice are expressed at dawn possibly due to repression by members of the PRR family (Fig. 6). PRR-dependent chloroplast gene regulation through *SIGE* expression may contribute to diurnal photosynthetic activity control and eventual productivity in these plants.

Collectively, our study implies that physiological processes controlled by the PRR transcription network, such as flowering time regulation, cell elongation, and chloroplast gene expression, are conserved in the three plants investigated in this study (Arabidopsis, poplar, and rice, Fig. 8). Although conservation of the PRR transcription networks is suggested by the *in silico* approaches, experimental approaches are also essential for further understanding of the PRR transcriptional network in an evolutionary context. In addition, characterizing other transcription networks controlled by clock-associated transcription factors such as LHY and LUX in other plants is a next step toward understanding the evolutionary development of the clock-transcription network.

## Methods

### Analysis of *PRR/TOC1* sequences

DNA sequences of members of the *PRR/TOC1* families in Arabidopsis, poplar and rice were obtained from Phytozome (https://phytozome.jgi.doe.gov/pz/portal.html)^64^. Since Arabidopsis *PRR5* in Phytozome contained an extra non-transcribed sequence upstream of the coding sequence^18^, the extra sequence was removed. No other *PRR/TOC1* sequences obtained from Phytozome were edited. DNA sequences of *PRR/TOC1* genes in maize and barley were obtained from NCBI (https://www.ncbi.nlm.nih.gov/search/) with the accession numbers as previously reported (NM_001154351 for *ZmPRR1/TOC1*, LOC100280240 for *ZmPRR37*, EU952116 for *ZmPRR73*, GRMZM2G135446 for *ZmPRR59*, NM_001158064 for *ZmPRR95*, and AY970701 for *Ppd-H1*)^61^. To create a phylogenic tree using the PR domain, PR domains in each PRR/TOC1 protein were searched as previously reported^26^. The evolutionary history was inferred using the neighbor-joining method^65^. The percentage of replicate trees in which the associated taxa clustered together in the bootstrap test (500 replicates) are shown next to the branches^66^. The tree is drawn to scale, with branch lengths in the same units as those of the evolutionary distances used to infer the phylogenetic tree. The evolutionary distances were computed using the Poisson correction method^67^ and are reported as the number of amino acid substitutions per site. Evolutionary analyses for PR domains were performed by MEGA7^68^. Repression and CCT domains were identified as reported previously^26,32^.

### Expression of *PRR/TOC1* genes

Expression of *PRR/TOC1* genes was surveyed in Diurnal 2.0, a database of array expression experiments. (http://diurnal.mocklerlab.org)^40^. The obtained expression data were used to make a heat map using Excel. Magenta, black, and green blocks show maximal expression levels, one-half expression levels, and no (zero) expression for each gene in plants growing under specific light conditions, respectively.

### Search of AtPRR5-target-like genes in poplar and rice

Arabidopsis PRR5-target genes^21^ were used as queries to search Phytozome for genes having similarity to PRR5-target genes in poplar and rice. The top two proteins having sequence similarities to each query were obtained. Some queries had only one or no homologous proteins (see the Results section for details). If identical sequences were found by searching with different queries, the two sequences were handled as a single sequence. The gene datasets are shown in Supplementary Tables 2 and 3.

### Phase-enrichment analysis of AtPRR5-target-like genes

Phase-enrichment analyses of PRR5-target and PRR-target-like genes were conducted using Phaser with a correction cutoff value of 0.7 (http://phaser.mocklerlab.org).

### Elucidation of enriched DNA sequences in upstream regions of AtPRR5-target-like genes

Upstream regions of AtPRR5-target-like gene coding sequences were obtained from PhytoMine in Phytozome (https://phytozome.jgi.doe.gov/phytomine/). Two-thousand base pairs of the 5′-upstream regions of these genes were analyzed in MEME (http://meme-suite.org/tools/meme)^69^. For MEME analysis of poplar AtPRR5-target-like genes, the following parameters were used. Background, A order-0 background generated from the supplied sequences. Discovery mode, Classic: optimizes the E-value of the motif information content. Site Distribution, Any number of repetitions (of a contributing motif site per sequence). Motif count, Searching for 10 motifs. Motif Width, Between 6 and 12 nucleotides wide (inclusive). The command line of the MEME analysis for poplar gene promoters was: meme input.fasta -o output_folder -dna -oc -mod anr -nmotifs 10 -minw 6 -maxw 12 -objfun classic -minsites 2 -maxsites 420 -revcomp -markov_order 0. The command line of the MEME for rice AtPRR5-target-like genes was: meme input.fasta -o output_folder -objfun classic -dna -mod anr -nmotifs 20 -evt 0.05 -minw 6 -maxw 12 -markov_order 1. The upstream regions of AtPRR5-target-like genes in poplar and rice are shown in Supplementary Datasets 1 and 2.

### Expression of AtPRR5-target-like genes encoding transcription factors

Expression of AtPRR5-target-like genes encoding transcription factors was surveyed using the DIURNAL database, and heat maps were generated as described above. Expression of rice AtPRR5-target-like TF genes was also surveyed in the Rice-X-Pro database (http://ricexpro.dna.affrc.go.jp)^70^, and a heat map was generated.

## Supplementary information

Supplementary Information

Dataset 1

Dataset 2
