# Supplementary material for: Evolutionary Insight into the Clock-Associated PRR5 Transcriptional Network of Flowering Plants
Source: Sci Rep. 2019 Feb 27;9:2983. doi: 10.1038/s41598-019-39720-2 (PMC6393427; doi:10.1038/s41598-019-39720-2)
Supplement: Supplementary file 1 — Supplementary Information [file 41598_2019_39720_MOESM1_ESM.pdf]

Supplementary Information

Evolutionary Insight into the Clock-Associated PRR5 Transcriptional Network of Flowering Plants

Yosuke Toda[1,2], Toru Kudo[3], Toshinori Kinoshita[2,4], Norihito Nakamichi*[2,4].

1. Precursory Research for Embryonic Science and Technology, Japan Science and Technology Agency, Kawaguchi, Saitama 332-0022, Japan.
2. Institute of Transformative Bio-molecules, Nagoya University, Furo-cho, Chikusa, Nagoya 464-8602, Japan.
3. Metabologenomics, Inc., 246-2 Mizukami Kakuganji, Tsuruoka, Yamagata 997-0052, Japan
4. Graduate School of Sciences, Nagoya University, Furo-cho, Chikusa, Nagoya 464-8602, Japan.
* corresponding author. , nnakamichi@itbm.nagoya-u.ac.jp

Content

Supplementary Figure 1. Amino acid sequence alignment of PRR proteins…………………………………2
Supplementary Figure 2. Expression of *AtPRR* genes in Diurnal……………………………………………..3
Supplementary Figure 3. Expression of homologues of *LHY* and *GI* in poplar and rice…………………...4
Supplementary Figure 4. The strategy for analyzing the possible PRR-transcription network…………….5
Supplementary Figure 5. Repeated sequences found by MEME analyses
of upstream regions of PRR5-target-like genes…………………………………6
Supplementary Figure 6. Ara-BOX-cis analysis of AtPRR5-target genes….. ………………………………7
Supplementary Table 1. Growth conditions used in the Diurnal database..…………………………………8
Supplementary Table 2. PRR5-target like genes in poplar……………………………………………………9
Supplementary Table 3. PRR5-target like genes in rice ………………………………………………… .10

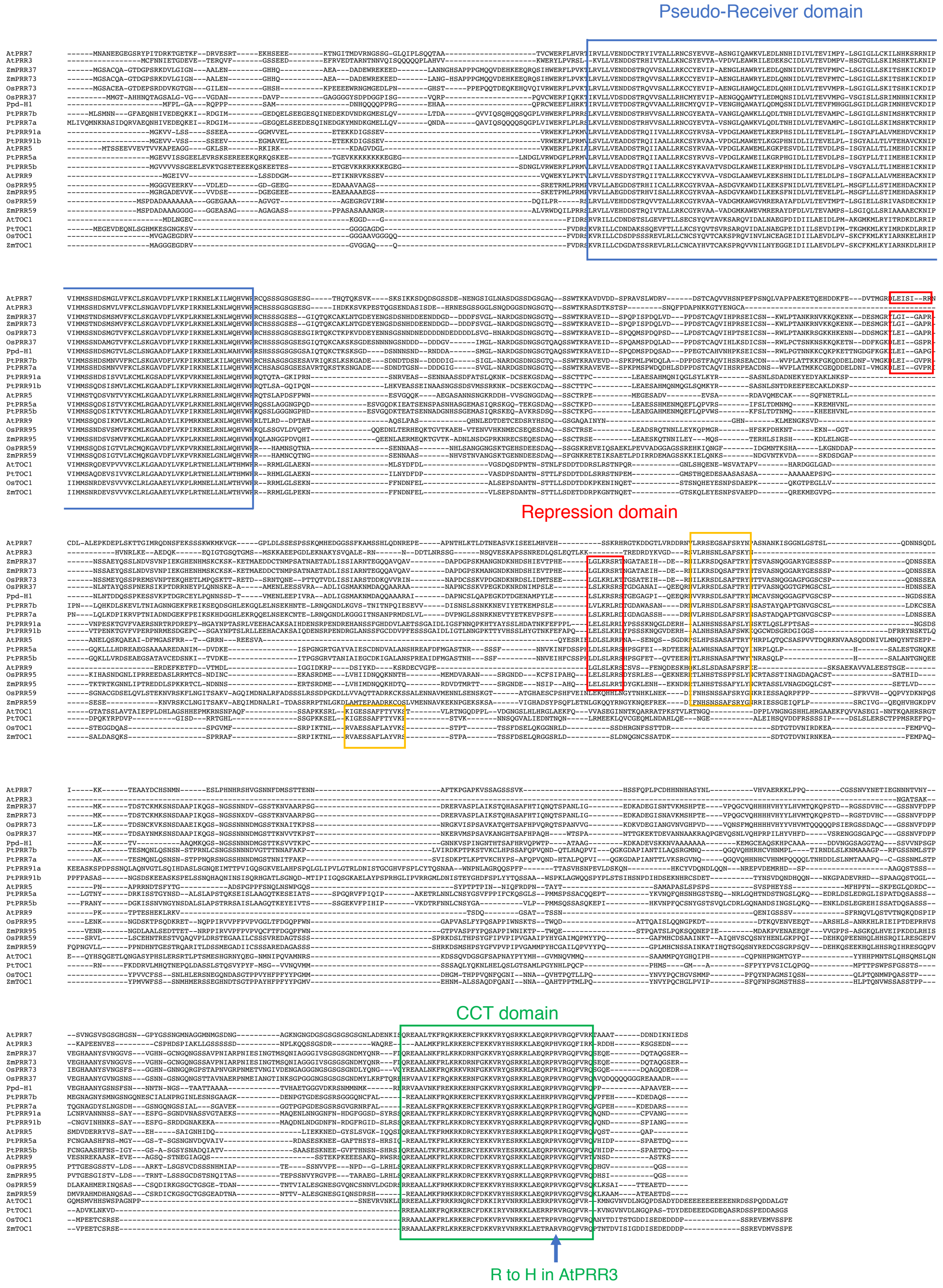

Supplementary Figure 1. Amino acid sequence alignment of PRR proteins.
The blue box identifies the pseudo-receiver domain. Red and orange boxes are conserved regions of the repression domain. OsPRR95, ZmPRR95, and AtPRR3 lack the red region. The green box identifies the CCT domain.

a

Signal Intensity

0 Half Max

Time (h)

	0	4	8	12	16	20	0	4	8	12	16	20
longday	0	4	8	12	16	20	0	4	8	12	16	20
LDHH_SM, LDHH_ST	0	4	8	12	16	20	0	4	8	12	16	20
shortday, COL_SD, LER_SD	0	4	8	12	16	20	0	4	8	12	16	20

Gene	Condition												
AtPRR9	longday	5	99	13	5	5	6	4	129	30	4	5	6
	LDHH_SM	5	429	26	7	5	5	5	325	34	7	5	5
	LDHH_ST	4	130	8	5	5	5	5	200	6	4	5	5
	shortday	7	225	12	4	4	5	7	254	12	5	5	5
	COL_SD	6	366	5	4	4	4	6	386	4	4	4	4
	LER_SD	10	284	4	4	4	4	11	282	4	5	3	4
AtPRR7	longday	9	165	530	139	23	7	7	225	697	126	15	5
	LDHH_SM	9	560	794	208	5	9	10	563	689	246	8	6
	LDHH_ST	9	436	411	74	6	5	9	501	598	45	6	6
	shortday	145	748	746	24	12	11	182	554	886	36	8	11
	COL_SD	14	363	298	7	6	8	13	329	311	6	6	8
	LER_SD	228	383	452	77	8	90	223	411	487	72	7	98
AtPRR5	longday	6	8	17	9	9	7	6	7	12	10	10	8
	LDHH_SM	9	6	1198	29	6	13	7	5	798	14	8	19
	LDHH_ST	5	5	487	18	9	8	5	6	617	26	13	13
	shortday	6	9	56	7	8	7	5	8	20	7	8	7
	COL_SD	9	11	365	13	26	16	8	11	396	14	28	18
	LER_SD	9	11	30	11	12	9	9	11	28	10	11	9
AtPRR3	longday	9	8	87	283	63	17	10	6	93	285	94	27
	LDHH_SM	11	8	202	558	26	15	14	7	281	599	28	16
	LDHH_ST	6	6	76	146	9	9	6	4	75	143	13	8
	shortday	7	8	226	101	24	18	6	9	242	108	26	25
	COL_SD	3	7	189	37	17	12	4	6	207	39	18	12
	LER_SD	6	7	224	121	45	46	6	7	215	119	42	51
AtTOC1	longday	55	29	292	645	411	141	104	31	265	639	403	218
	LDHH_SM	40	17	403	566	106	251	32	22	321	711	113	267
	LDHH_ST	26	19	357	585	157	167	32	16	426	484	117	197
	shortday	30	56	812	397	425	184	34	78	832	436	355	269
	COL_SD	17	22	538	281	300	219	18	22	515	268	324	218
	LER_SD	47	37	698	436	377	286	43	36	755	405	355	288

b

Time (h)

	0	4	8	12	16	20	24	28	32	36	40	44
LL23_LDHH	0	4	8	12	16	20	24	28	32	36	40	44
DD_DDHC	0	4	8	12	16	20	24	28	32	36	40	44

Gene	Condition												
AtPRR9	LL23_LDHH	14	63	8	6	6	8	25	45	10	7	7	9
	DD_DDHC	5	8	5	4	5	4	4	8	7	6	5	5
AtPRR7	LL23_LDHH	37	244	278	10	9	10	109	334	184	12	10	17
	DD_DDHC	8	49	24	12	7	5	7	36	53	21	15	9
AtPRR5	LL23_LDHH	6	14	113	13	13	9	8	48	63	18	15	10
	DD_DDHC	6	5	16	10	8	5	5	6	19	22	15	8
AtPRR3	LL23_LDHH	11	11	201	87	37	21	16	25	145	102	35	15
	DD_DDHC	5	5	12	21	10	20	10	7	13	12	26	21
AtTOC1	LL23_LDHH	16	34	490	362	204	69	17	110	468	461	168	55
	DD_DDHC	31	26	69	137	119	148	82	42	106	190	302	231

Supplementary Figure 2. Expression of *AtPRR* genes in Diurnal.
Expression of *AtPRR* genes in Diurnal 2.0 (a, light-dark conditions, b, constant light or constant dark conditions).

Signal Intensity

0 Half Max

a

Time (h)	0	4	8	12	16	20	24	28	32	36	40	44
Condition						*PtLHY1*						
LDHH	337	277	38	20	15	174	369	272	70	20	24	189
LDHC	405	262	76	54	82	223	362	324	90	46	83	264
						PtLHY2						
LDHH	11471	6104	349	125	120	6005	13874	6229	807	171	164	6388
LDHC	8405	4210	485	241	567	4995	7546	4565	738	141	668	5062
						PtGIL						
LDHH	73	633	1174	1893	236	133	109	608	1861	1547	564	199
LDHC	798	765	1137	1541	1838	1012	579	1004	919	1298	1509	722
						PtGI						
LDHH	25	604	2108	2195	145	41	49	727	2372	1760	268	55
LDHC	808	1059	1422	1625	1978	1209	608	1625	1504	1803	1662	1923

b

Time (h)	0	4	8	12	16	20	24	28	32	36	40	44
Condition						*PtLHY1*						
LLHH_LLHC	227	159	211	178	171	238	253	311	278	215	198	274
LLHH_LDHH	214	172	197	185	125	181	175	168	152	213	190	128
LLHH_LDHC	268	252	222	243	189	396	174	289	183	287	314	276
LLHC	292	186	93	56	102	302	187	130	97	118	102	234
						PtLHY2						
LLHH_LLHC	1448	898	1448	898	1218	1448	1700	1930	1645	1303	1201	1764
LLHH_LDHH	1321	1005	1099	1727	785	1057	1044	1007	714	1327	1087	775
LLHH_LDHC	2335	1407	1263	1471	1098	2576	909	1596	1142	1653	2059	1361
LLHC	4836	2332	802	340	1016	5664	3464	1859	677	1116	898	3239
						PtGIL						
LLHH_LLHC	1850	2012	1595	1326	1372	2183	2129	2877	2170	2272	2053	1851
LLHH_LDHH	1065	1331	1831	1381	1219	1497	1336	1090	947	1097	1579	940
LLHH_LDHC	3132	1830	1456	2068	1317	2556	1010	1632	1300	1573	1499	1707
LLHC	1499	1734	1773	927	1263	2554	1409	1309	1232	1599	963	986
						PtGI						
LLHH_LLHC	2985	2867	2637	2051	3010	2919	3364	4028	3540	2867	2578	2745
LLHH_LDHH	1430	1253	1276	1304	1321	1244	1088	1085	1091	1297	1269	1073
LLHH_LDHC	3405	1779	1495	2203	1659	3389	1421	2366	1361	1670	1735	1870
LLHC	3218	2125	2302	1486	2423	4708	2234	1568	1793	1933	1910	2404

c

Time (h)	0	4	8	12	16	20	0	4	8	12	16	20
Condition						*OsLHY*						
LDHH	12,695	9,045	1,624	255	294	3,042	12,212	8,494	1,236	215	237	3,145
LDHC	13,153	8,070	910	278	671	10,126	14,150	7,088	939	207	726	9,292
						OsGI						
LDHH	20	1,688	4,114	6,835	755	111	27	1,498	4,117	4,283	547	72
LDHC	714	2,406	6,593	7,552	3,883	1,379	716	2,335	5,752	6,897	4,230	1,721

d

Time (h)	0	4	8	12	16	20	24	28	32	36	40	44
Condition						*OsLHY*						
LLHH_LLHC	2,709	3,364	4,268	4,665	4,372	2,544	2,297	4,481	3,154	4,024	5,424	3,277
LLHH_LDHH	777	1,649	1,693	2,365	1,571	1,512	1,307	1,526	1,842	1,786	1,940	1,953
LLHH_LDHC	1,199	1,940	1,026	1,447	1,630	1,136	757	779	2,099	507	778	929
LLHC	7,448	1,335	504	313	2,858	11,665	11,330	2,059	378	244	3,865	8,313
						OsGI						
LLHH_LLHC	6,007	4,625	4,640	4,687	4,810	6,087	6,748	5,317	4,374	4,597	4,447	4,002
LLHH_LDHH	4,740	6,186	6,007	6,115	5,875	5,777	5,682	5,694	5,962	6,232	6,077	6,063
LLHH_LDHC	5,988	6,436	5,399	5,623	6,820	5,993	6,025	5,314	6,811	4,405	5,502	5,807
LLHC	5,469	6,250	9,162	6,819	6,611	6,865	6,231	5,595	9,321	6,223	6,677	4,105

Supplementary Figure 3. Expression of homologues of *LHY* and *GI* in poplar and rice. Expression of poplar *LHY* or *GI* homologues (panels a and b) and rice *LHY* and *GI* homologues (panels c and d). Panels a and c summarize experiments conducted under light-dark conditions, and those in panels b and d show heat maps from constant light conditions.

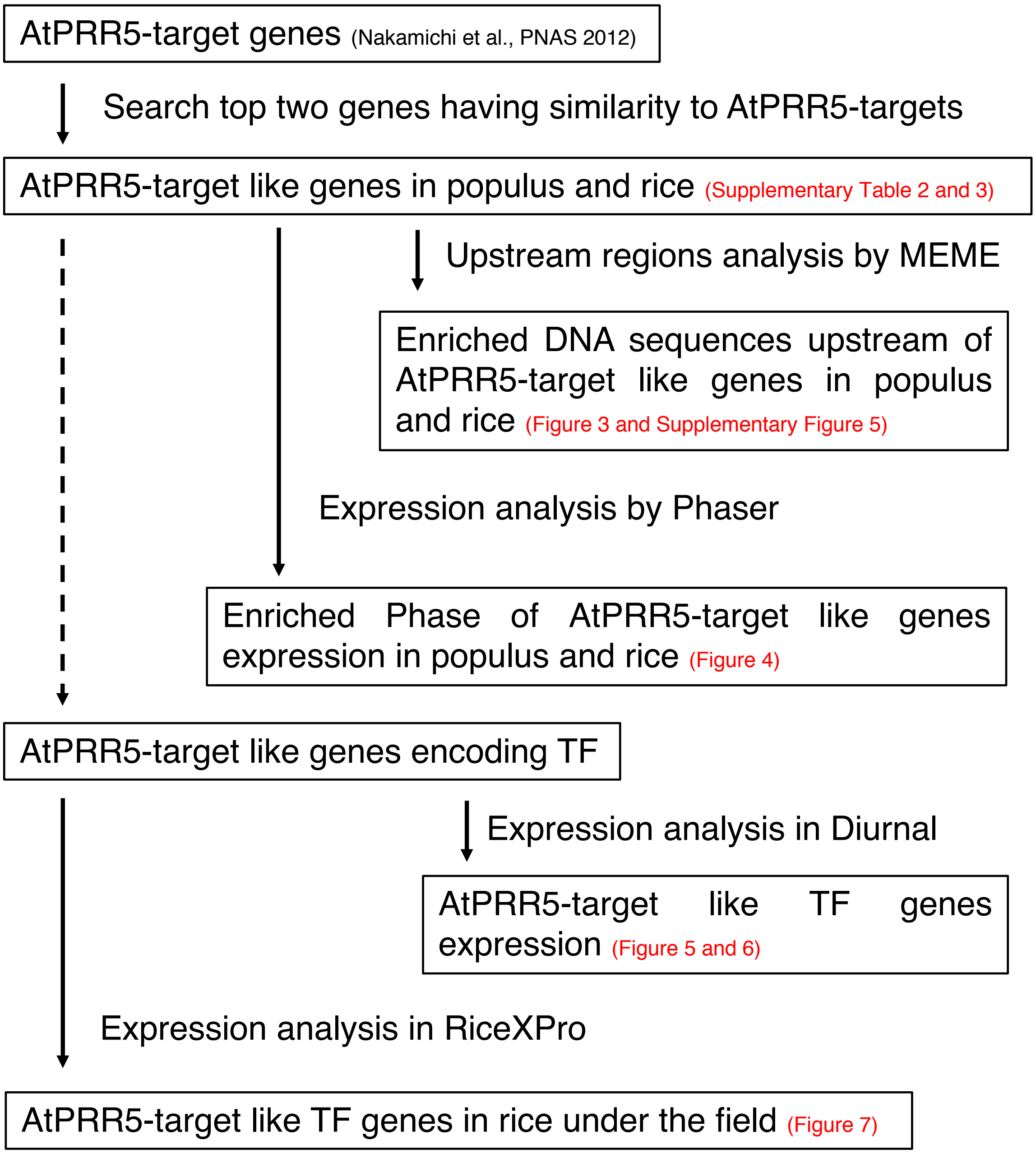

Supplementary Figure 4. The strategy for analyzing the possible PRR-transcription network.

Poplar

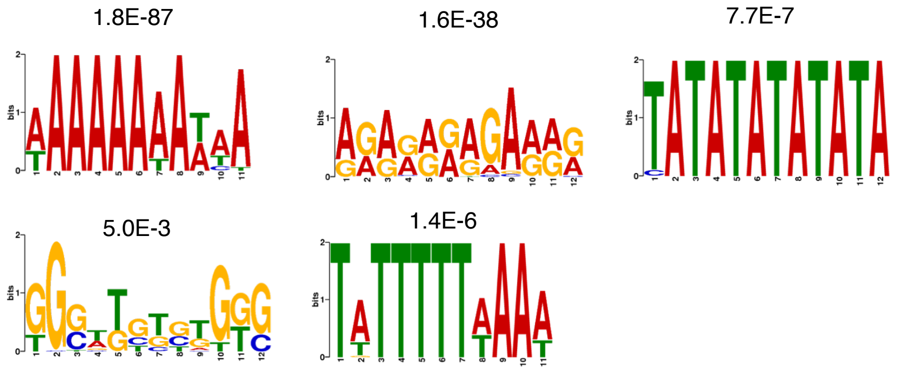

Rice

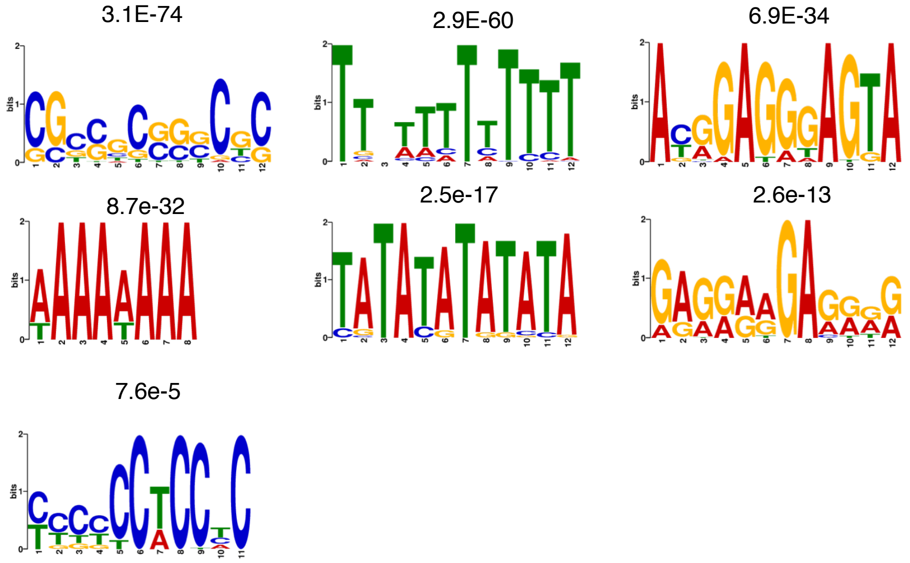

Supplementary Figure 5. Repeated sequences found by MEME analyses of upstream regions of PRR5-target-like genes.

Common motifs found in the upstream regions of PRR5-target-like genes in poplar and rice. Only repeated sequences are shown. Non-repeated sequences are presented in Fig. 3.

TFs predictive of gene expression
AT4G34000 (ABF3) count:8/44
AT3G23210 (bHLH34) count:5/80
AT1G19490 count:5/49
AT3G59060 (PIL6) count:5/21
AT1G49720 (ABF1) count:4/52
AT1G13600 (bZIP58) count:3/17
AT5G24800 (BZIP9) count:3/17
AT5G11260 (HY5) count:3/31
AT1G62975 count:3/45
AT2G46270 (GBF3) count:2/16
AT5G28770 (BZO2H3) count:2/42
AT2G43060 (IBH1) count:1/10
AT3G17100 count:1/49
AT1G59640 (BPEP) count:1/13
AT5G61270 (PIF7) count:1/71
AT2G43010 (PIF4) count:1/4
AT1G35460 (FBH1) count:1/6
AT4G36730 (GBF1) count:1/49
AT1G02340 (HFR1) count:1/15
AT4G17880 (MYC4) count:1/44

Supplementary Figure 6. Ara-BOX-cis analysis of At-PRR5-targets.
At-PRR5-target genes were submitted in “Set of Genes” in Ara-BOX-cis database.

Supplementary Table 1. Growth conditions used in the Diurnal database.

Arabidopsis. Reference, Michael et al., PLoS Genet., 2008.

Name	Light and temperature	Age	Acc.	Media	Tissue	Light intensity
Long Day	16h L/ 8h D, constant 22˚C	7day	Ler	agar, 3% suc	seedling	90uE
COL LDHH	12h L/ 12h D, constant 22˚C	7day	Col-0	agar, 3% suc	seedling	120uE
LDHH-SM	12h L/ 12h D, constant 22˚C	29day	Col-0	soil	leaf	180uE
LDHH-ST	12h L/ 12h D, constant 22˚C	35day	Col-0	soil	leaf	130uE
Short Day	8h L/ 16h D, constant 22˚C	7day	Ler	agar, 3% suc	seedling	180uE
COL SD	8h L/ 16h D, constant 22˚C	7day	Col-0	agar, no suc	seedling	100uE
LER SD	8h L/ 16h D, constant 22˚C	7day	Ler	agar, no suc	seedling	100uE
LDHC	12h L, 22˚C / 12h D, 12˚C	7day	Col-0	agar, no suc	seedling	100uE
LLHC	12h 22˚C / 12h 12˚C, constant light	7day	Col-0	agar, no suc	seedling	100uE
LL12_LDHH	constant light, constant 22˚C	7day	Col-0	agar, 3% suc	seedling	100uE
LL23_LDHH	constant light, constant 22˚C	8day	Col-0	agar, 3% suc	seedling	60uE
LL_LDHC	constant light, constant 22˚C	9day	Col-0	agar, no suc	seedling	100uE
LL_LLHC	constant light, constant 22˚C	9day	Col-0	agar, no suc	seedling	100uE
DD_DDHC	constant dark, constant 22˚C	8day	Col-0	agar, 3% suc	seedling	0uE

Poplar. Reference, Filichkin et al., PLoS One, 2011.

Name	Light and temperature	Age	Acc.	Media	Tissue	Light intensity
LDHH	12h L/ 12h D, constant 25˚C	3month from fresh cuttings	Populus trichocarpa, clone Nisqually-1	not described	leaves including petioles	700μE
LDHC	12h L, 25˚C / 12h D, 12˚C	3month from fresh cuttings	Populus trichocarpa, clone Nisqually-1	not described	leaves including petioles	700μE
LLHH_LLHC	constant light, constant 25˚C	3month from fresh cuttings	Populus trichocarpa, clone Nisqually-1	not described	leaves including petioles	700μE
LLHH_LDHH	constant light, constant 25˚C	3month from fresh cuttings	Populus trichocarpa, clone Nisqually-1	not described	leaves including petioles	700μE
LLHH_LDHC	constant light, constant 25˚C	3month from fresh cuttings	Populus trichocarpa, clone Nisqually-1	not described	leaves including petioles	700μE
LLHC	12h 25˚C / 12h 12˚C, constant light	3month from fresh cuttings	Populus trichocarpa, clone Nisqually-1	not described	leaves including petioles	700μE

Rice. Reference, Filichkin et al., PLoS One, 2011.

Name	Light and temperature	Age	Acc.	Media	Tissue	Light intensity
LDHH	12h L/ 12h D, constant 31˚C	3month	ssp. Japonica, cv. Nipponbare 1	not described	leavs and stems	1000uE
LDHC	12h L, 31˚C / 12h D, 20˚C	3month	ssp. Japonica, cv. Nipponbare 1	not described	leavs and stems	1000uE
LLHH_LLHC	constant light, constant 31˚C	3month	ssp. Japonica, cv. Nipponbare 1	not described	leavs and stems	1000uE
LLHH_LDHH	constant light, constant 31˚C	3month	ssp. Japonica, cv. Nipponbare 1	not described	leavs and stems	1000uE
LLHH_LDHC	constant light, constant 31˚C	3month	ssp. Japonica, cv. Nipponbare 1	not described	leavs and stems	1000uE
LLHC	12h 31˚C / 12h 20˚C, constant light	3month	ssp. Japonica, cv. Nipponbare 1	not described	leavs and stems	1000uE

Supplementary Table 2. PRR5-target like genes in poplar.

AGI code	annotation	ID	ID2	score (Dual Affine Smith-Waterman Alignment score)	similarity (%) (Fraction of identical positively scoring residues in alignment)
AT1G01060	LHY (LATE ELONGATED HYPO(	Potri.002G180800.1	POPTR_0002s18190	1058	62.6
AT1G01060	LHY (LATE ELONGATED HYPO(	Potri.014G106800.1	POPTR_0014s10260	1114	59.7
AT1G01070	nodulin MtN21 family protein	Potri.001G453000.1	POPTR_0001s45730	916	60.3
AT1G01070	nodulin MtN21 family protein	Potri.011G149000.1	POPTR_0011s15200	905	57.5
AT1G01520	myb family transcription factor	Potri.002G163100.2	POPTR_0002s16460	777	62
AT1G01520	myb family transcription factor	Potri.014G089300.1	POPTR_0014s08520	819	71.4
AT1G02340	HFR1 (LONG HYPOCOTYL IN F	Potri.005G207200.6	POPTR_0005s22870	215	26.7
AT1G02340	HFR1 (LONG HYPOCOTYL IN F	Potri.013G001300.1	POPTR_0013s00310	232	32.5
AT1G09350	ATGOLS3 (ARABIDOPSIS THA	Potri.010G150400.1	POPTR_0010s16020	1478	78.7
AT1G14280	PKS2 (PHYTOCHROME KIN/	Potri.015G062600.1	POPTR_0015s07390	166	34.8
AT1G18330	RVE7	Potri.012G038300.3	POPTR_0012s03530	488	51.9
AT1G18330	RVE7	Potri.015G030400.1	POPTR_0015s05430	492	38.4
AT1G29440	auxin-responsive family prot	Potri.017G043600.1	POPTR_0017s07110	360	73
AT1G32900	starch synthase, putative	Potri.001G449500.1	POPTR_0001s45360	2660	81.1
AT1G32900	starch synthase, putative	Potri.011G152200.1	POPTR_0011s15540	2600	79.7
AT1G48100	glycoside hydrolase family 2	Potri.008G100500.1	POPTR_0008s09990	1963	80.2
AT1G48100	glycoside hydrolase family 2	Potri.010G152000.1	POPTR_0010s16180	1898	70.3
AT1G64780	ATAMT1;2 (AMMONIUM TR/	Potri.019G023600.1	POPTR_0019s04030	2231	77.6
AT1G64780	ATAMT1;2 (AMMONIUM TR/	Potri.013G049600.1	POPTR_0013s04710	2100	74.7
AT1G68440	unknown protein	Potri.008G121700.1	POPTR_0008s12090	353	51
AT1G68440	unknown protein	Potri.010G123600.1	POPTR_0010s13350	444	55.2
AT1G69160	unknown protein	Potri.010G154200.1	POPTR_0010s16385	219	52.3
AT1G73480	hydrolase, alpha/beta fold fa	Potri.012G041800.1	POPTR_0012s03860	1434	66.7
AT1G73480	hydrolase, alpha/beta fold fa	Potri.015G031900.2	POPTR_0015s05290	1421	65.2
AT1G75180	Erythronate-4-phosphate de	Potri.002G260900.1	POPTR_0002s26240	1058	77.8
AT1G75180	Erythronate-4-phosphate de	Potri.009G115800.2	POPTR_0009s11860	926	68.3
AT1G78070	WD-40 repeat family protein	Potri.005G168600.1	POPTR_0005s16680	1957	81.1
AT1G78070	WD-40 repeat family protein	Potri.002G094100.1	POPTR_0002s09470	1969	82.6
AT2G22240	inositol-3-phosphate synthase	Potri.005G078700.1	POPTR_0005s08050	2876	97.1
AT2G22240	inositol-3-phosphate synthase	Potri.007G089000.11	POPTR_0007s05810	2870	96.9
AT2G23120	unknown protein	Potri.005G145200.1	POPTR_0005s18870	146	33.7
AT2G24540	kelch repeat-containing F-b	Potri.006G193800.1	POPTR_0006s20830	846	55.4
AT2G24540	kelch repeat-containing F-b	Potri.018G007100.1	POPTR_0018s03590	1292	71.2
AT2G30520	RPT2 (ROOT PHOTOTROPIS	Potri.013G159000.1	POPTR_0013s15500	2445	82
AT2G30520	RPT2 (ROOT PHOTOTROPIS	Potri.019G131600.1	POPTR_0019s15220	2357	80.9
AT2G31380	STH (salt tolerance homologue)	Potri.017G028300.1	POPTR_0017s05700	878	74.8
AT2G41250	haloacid dehalogenase-like I	Potri.006G040800.2	POPTR_0006s03920	1193	73.8
AT2G41250	haloacid dehalogenase-like I	Potri.016G037600.1	POPTR_0016s03710	1216	74.5
AT2G47180	ATGOLS1 (ARABIDOPSIS THA	Potri.002G191600.1	POPTR_0002s19230	1760	87.8
AT3G09600	myb family transcription factor	Potri.016G083900.1	POPTR_0016s08470	945	65.4
AT3G09600	myb family transcription factor	Potri.006G133000.3	POPTR_0006s13530	960	64.1
AT3G19270	CYP707A4 (cytochrome P45(	Potri.004G140900.1	POPTR_0004s14820	2251	84.4
AT3G22840	ELIP1 (EARLY LIGHT-INDU(	Potri.008G158000.1	POPTR_0008s15780	565	74.9
AT3G47500	CDF3 (CYCLING DOF FACTOR	Potri.004G121800.1	POPTR_0004s12120	882	49.1
AT3G51895	SULTR3;1 (SULFATE TRANS	Potri.002G049500.1	POPTR_0002s05030	2982	86.6
AT3G54500	LNK2	Potri.003G200600.1	POPTR_0003s19960	910	49.9
AT3G54500	LNK2	Potri.001G025100.13	POPTR_0001s06240	836	46.8
AT3G54510	unknown protein	Potri.002G113800.1	POPTR_0002s11450	1226	52.5
AT3G54510	unknown protein	Potri.003G200900.1	POPTR_0003s19990	2496	71.5
AT3G55580	regulator of chromosome cor	Potri.010G199100.1	POPTR_0010s20660	1788	75.6
AT3G55580	regulator of chromosome cor	Potri.008G059800.3	POPTR_0008s06020	1815	77.3
AT3G59060	PIL6 (PHYTOCHROME-INTERA	Potri.002G055400.3	POPTR_0002s05630	522	52
AT3G62690	ATL5 (Arabidopsis T?xicos e	Potri.T030300.1	POPTR_0010s01400	387	56.8
AT4G25470	CBF2 (FREEZING TOLERANCE	Potri.001G110700.1	POPTR_0001s08720	569	56.5
AT4G25480	DREB1A (DEHYDRATION RESP	Potri.015G136400.1	POPTR_0015s13830	580	65.3
AT4G25490	CBF1 (C-REPEAT/DRE BINDIN	Potri.012G134100.1	POPTR_0012s13870	589	63.4
AT4G36040	DNAJ heat shock N-termina	Potri.002G020700.1	POPTR_0002s02220	316	52.2
AT4G36040	DNAJ heat shock N-termina	Potri.005G113100.1	POPTR_0005s11520	409	56.5
AT4G36700	cupin family protein	Potri.009G054700.1	POPTR_0009s05950	452	36.2
AT4G38860	auxin-responsive protein, pu	Potri.009G126000.1	POPTR_0009s12890	527	90.5
AT4G38860	auxin-responsive protein, pu	Potri.004G164400.1	POPTR_0004s17150	524	90.5
AT5G01820	ATSR1 (SERINE/THREONIN	Potri.016G133900.1	POPTR_0016s14140	1442	66.1
AT5G01820	ATSR1 (SERINE/THREONIN	Potri.016G133500.1	POPTR_0016s14100	1127	64.5
AT5G06980	LNK4	Potri.001G031800.5	POPTR_0001s06910	250	52.3
AT5G06980	LNK4	Potri.003G193800.3	POPTR_0003s19320	245	50.9
AT5G15850	COL1 (CONSTANS-LIKE 1); tra	Potri.017G107500.1	POPTR_0017s14410	965	69.3
AT5G15940	short-chain dehydrogenase/	Potri.001G453500.1	POPTR_0001s45770	1212	74.2
AT5G15940	short-chain dehydrogenase/	Potri.003G182000.3	POPTR_0003s18040	287	45.9
AT5G15950	adenosylmethionine decarbo:	Potri.017G108800.1	POPTR_0017s14280	1414	76.8
AT5G15950	adenosylmethionine decarbo:	Potri.010G133900.1	POPTR_0010s14390	1380	79
AT5G17300	RVE1	Potri.004G074300.5	POPTR_0004s07270	519	46
AT5G17310	UTP--glucose-1-phosphate	Potri.017G144700.1	POPTR_0017s01390	2324	93
AT5G17310	UTP--glucose-1-phosphate	Potri.004G074400.2	POPTR_0004s07280	2262	90.4
AT5G23730	EFO2, REPRESSOR OF UV-	Potri.012G140200.1	POPTR_0012s13290	1362	68.8
AT5G23730	EFO2, REPRESSOR OF UV-	Potri.015G143200.1	POPTR_0015s13210	1348	67.1
AT5G24120	SIGE (RNA polymerase sigm	Potri.012G031100.1	POPTR_0012s01430	1711	75.4
AT5G39660	CDF2 (CYCLING DOF FACTOR	Potri.017G084600.1	POPTR_0017s12080	933	51
AT5G39660	CDF2 (CYCLING DOF FACTOR	Potri.008G087800.1	POPTR_0008s08740	798	52.1
AT5G57660	zinc finger (B-box type) family p	Potri.006G173600.1	POPTR_0006s18760	993	68.7
AT5G57660	zinc finger (B-box type) family p	Potri.T094400.1	POPTR_0018s10510	881	64.2
AT5G59730	ATEXO70H7 (EXOCYST SUE	Potri.001G234600.1	POPTR_0001s24130	1619	65.9
AT5G59730	ATEXO70H7 (EXOCYST SUE	Potri.009G026900.1	POPTR_0009s03200	1688	65.9
AT5G62130	Per1-like protein-related	Potri.007G065800.1	POPTR_0007s08380	1371	73.9
AT5G62130	Per1-like protein-related	Potri.015G136600.1	POPTR_0015s13810	1356	72.2

Supplementary Table 3. PRR5-target like genes in rice.

AGI code	annotation	ID	score (Dual Affine Smith-Waterman Alignment score)	similarity (%) (Fraction of identical positively scoring residues in alignment)
AT1G01060	LHY (LATE ELONGATED HYPOCOT	LOC_Os08g06110	786	50.7
AT1G01070	nodulin MtN21 family protein	LOC_Os04g38430	684	54.5
AT1G01070	nodulin MtN21 family protein	LOC_Os02g01326	734	52.9
AT1G01520	myb family transcription factor	LOC_Os02g45670	740	59.2
AT1G01520	myb family transcription factor	LOC_Os06g45840	714	58.2
AT1G06040	STO (SALT TOLERANCE); transcrip	LOC_Os04g41560	530	58.1
AT1G18330	RVE7	LOC_Os04g49450	346	35.5
AT1G18330	RVE7	LOC_Os06g51260	339	40.1
AT1G29440	auxin-responsive family protein	LOC_Os09g37430	297	51.8
AT1G32900	starch synthase, putative	LOC_Os07g22930	2273	70.2
AT1G32900	starch synthase, putative	LOC_Os06g04200	2242	67.5
AT1G48100	glycoside hydrolase family 28 pı	LOC_Os05g20020	1652	60.2
AT1G48100	glycoside hydrolase family 28 pı	LOC_Os01g22590	1467	61.5
AT1G64780	ATAMT1;2 (AMMONIUM TRANS	LOC_Os04g43070	2073	73
AT1G64780	ATAMT1;2 (AMMONIUM TRANS	LOC_Os02g40730	1881	67.5
AT1G69570	Dof-type zinc finger domain-contai	LOC_Os03g07360	467	46.1
AT1G73480	hydrolase, alpha/beta fold famil	LOC_Os09g23150	1173	51.8
AT1G73480	hydrolase, alpha/beta fold famil	LOC_Os03g51010	1121	49.2
AT1G75180	Erythronate-4-phosphate dehyd	LOC_Os03g12700	349	41.6
AT1G75180	Erythronate-4-phosphate dehyd	LOC_Os03g56420	771	66.3
AT1G78070	WD-40 repeat family protein	LOC_Os03g26870	1651	69.7
AT1G78070	WD-40 repeat family protein	LOC_Os08g38880	1523	69.9
AT2G22240	inositol-3-phosphate synthase isoz	LOC_Os03g09250	2788	96.1
AT2G22240	inositol-3-phosphate synthase isoz	LOC_Os10g22450	2653	93.1
AT2G24540	kelch repeat-containing F-box f	LOC_Os07g05880	775	55.1
AT2G24540	kelch repeat-containing F-box f	LOC_Os06g44500	487	40.1
AT2G30520	RPT2 (ROOT PHOTOTROPISM :	LOC_Os11g02610	1160	55.3
AT2G30520	RPT2 (ROOT PHOTOTROPISM :	LOC_Os12g02530	1166	55.5
AT2G31380	STH (salt tolerance homologue); tra	LOC_Os02g39360	487	56.7
AT2G41250	haloacid dehalogenase-like hydr	LOC_Os07g46520	1138	70.7
AT2G41250	haloacid dehalogenase-like hydr	LOC_Os08g07300	544	40.3
AT2G43010	PIF4 (PHYTOCHROME INTERACTIN	LOC_Os12g41650	279	27.9
AT2G47180	ATGOLS1 (ARABIDOPSIS THALIAI	LOC_Os03g20120	1572	77.6
AT2G47180	ATGOLS1 (ARABIDOPSIS THALIAI	LOC_Os07g48830	1562	77.3
AT3G09600	myb family transcription factor	LOC_Os06g01670	671	58.7
AT3G19270	CYP707A4 (cytochrome P450, fa	LOC_Os08g36860	1717	70.7
AT3G22840	ELIP1 (EARLY LIGHT-INDUCAB	LOC_Os01g14410	362	38.5
AT3G22840	ELIP1 (EARLY LIGHT-INDUCAB	LOC_Os07g08160	415	42.1
AT3G47500	CDF3 (CYCLING DOF FACTOR 3); [	LOC_Os01g15900	617	48.9
AT3G51895	SULTR3;1 (SULFATE TRANSPO	LOC_Os03g06520	2672	78
AT3G51895	SULTR3;1 (SULFATE TRANSPO	LOC_Os01g52130	2138	69.3
AT3G54510	unknown protein	LOC_Os12g43720	1318	52.7
AT3G54510	unknown protein	LOC_Os01g72210	2071	66.4
AT3G55580	regulator of chromosome conder	LOC_Os01g62810	1718	71.1
AT3G55580	regulator of chromosome conder	LOC_Os05g38270	1618	66.6
AT3G62690	ATL5 (Arabidopsis T?xicos en L	LOC_Os02g57460	260	20.2
AT4G25480	DREB1A (DEHYDRATION RESPON:	LOC_Os04g48350	393	52.3
AT4G25480	DREB1A (DEHYDRATION RESPON:	LOC_Os02g45450	389	58.8
AT4G36040	DNAJ heat shock N-terminal dc	LOC_Os01g01160	195	35.4
AT4G36040	DNAJ heat shock N-terminal dc	LOC_Os08g43490	224	32.9
AT4G36700	cupin family protein	LOC_Os03g46100	238	31.4
AT4G38860	auxin-responsive protein, putati	LOC_Os02g24700	335	68.6
AT4G38860	auxin-responsive protein, putati	LOC_Os06g04590	254	61.9
AT5G01820	ATSR1 (SERINE/THREONINE P	LOC_Os01g10890	1169	62.7
AT5G01820	ATSR1 (SERINE/THREONINE P	LOC_Os05g26820	1152	63.1
AT5G15850	COL1 (CONSTANS-LIKE 1); transc	LOC_Os06g16370	575	56.6
AT5G15850	COL1 (CONSTANS-LIKE 1); transc	LOC_Os02g39710	396	47
AT5G15940	short-chain dehydrogenase/redu	LOC_Os09g17750	830	54.7
AT5G15940	short-chain dehydrogenase/redu	LOC_Os11g07930	326	45.1
AT5G15950	adenosylmethionine decarboxyla	LOC_Os04g42090	1047	64.9
AT5G15950	adenosylmethionine decarboxyla	LOC_Os04g42095	1047	64.9
AT5G17300	RVE1	LOC_Os02g46030	391	20.4
AT5G17310	UTP--glucose-1-phosphate uric	LOC_Os09g38030	2325	93.4
AT5G17310	UTP--glucose-1-phosphate uric	LOC_Os02g02560	2315	91.1
AT5G23730	EFO2, REPRESSOR OF UV-B P	LOC_Os02g53140	557	45.7
AT5G24120	SIGE (RNA polymerase sigma sı	LOC_Os05g50930	1226	58
AT5G39660	CDF2 (CYCLING DOF FACTOR 2); [	LOC_Os07g48570	755	45.5
AT5G43150	unknown protein	LOC_Os04g51410	150	38.5
AT5G43150	unknown protein	LOC_Os03g10860	200	38.5
AT5G54470	zinc finger (B-box type) family prote	LOC_Os06g19444	184	21.9
AT5G54470	zinc finger (B-box type) family prote	LOC_Os08g08120	300	21.9
AT5G57660	zinc finger (B-box type) family prote	LOC_Os04g42020	514	39.2
AT5G59730	ATEXO70H7 (EXOCYST SUBUN	LOC_Os04g31330	826	40.1
AT5G59730	ATEXO70H7 (EXOCYST SUBUN	LOC_Os02g30230	815	38.6
AT5G62130	Per1-like protein-related	LOC_Os10g38030	1027	61.2
AT5G62130	Per1-like protein-related	LOC_Os05g13330	1128	64.1
AT5G64170	dentin sialophosphoprotein-relat	LOC_Os03g27019	419	38.1
